# Increased regional ventilation as early imaging marker for future disease progression of interstitial lung disease: a feasibility study

**DOI:** 10.1007/s00330-022-08702-w

**Published:** 2022-03-31

**Authors:** Sarah C. Scharm, Cornelia Schaefer-Prokop, Moritz Willmann, Jens Vogel-Claussen, Lars Knudsen, Danny Jonigk, Jan Fuge, Tobias Welte, Frank Wacker, Antje Prasse, Hoen-oh Shin

**Affiliations:** 1 Institute of Diagnostic and Interventional Radiology, Hannover Medical School, Carl-Neuberg-Str.1, 30625 Hannover, Germany; 2 Department of Radiology, Radboud University, Nijmegen, The Netherlands; 3 Department of Radiology, Meander Medical Center, Amersfoort, The Netherlands; 4 Biomedical Research in Endstage and Obstructive Lung Disease Hannover (BREATH), German Center for Lung Research, Hannover, Germany; 5 Institute of Functional and Applied Anatomy, Hannover Medical School, Hannover, Germany; 6 Institute of Pathology, Hannover Medical School, Hannover, Germany; 7 Department of Respiratory Medicine, Hannover Medical School, Hannover, Germany

**Keywords:** Computed tomography, volume, Image analysis, computer-assisted, Lung volume measurements, Pulmonary ventilation, Diagnostic techniques, respiratory system

## Abstract

**Objectives:**

Idiopathic pulmonary fibrosis (IPF) is a disease with a poor prognosis and a highly variable course. Pathologically increased ventilation—accessible by functional CT—is discussed as a potential predecessor of lung fibrosis. The purpose of this feasibility study was to investigate whether increased regional ventilation at baseline CT and morphological changes in the follow-up CT suggestive for fibrosis indeed occur in spatial correspondence.

**Methods:**

In this retrospective study, CT scans were performed at two time points between September 2016 and November 2020. Baseline ventilation was divided into four categories ranging from low, normal to moderately, and severely increased (C1–C4). Correlation between baseline ventilation and volume and density change at follow-up was investigated in corresponding voxels. The significance of the difference of density and volume change per ventilation category was assessed using paired *t*-tests with a significance level of *p* ≤ 0.05. The analysis was performed separately for normal (NAA) and high attenuation areas (HAA).

**Results:**

The study group consisted of 41 patients (73 ± 10 years, 36 men). In both NAA and HAA, significant increases of density and loss of volume were seen in areas of severely increased ventilation (C4) at baseline compared to areas of normal ventilation (C2, *p* < 0.001). In HAA, morphological changes were more heterogeneous compared to NAA.

**Conclusion:**

Functional CT assessing the extent and distribution of lung parenchyma with pathologically increased ventilation may serve as an imaging marker to prospectively identify lung parenchyma at risk for developing fibrosis.

**Key Points:**

• *Voxelwise correlation of serial CT scans suggests spatial correspondence between increased ventilation at baseline and structural changes at follow-up.*

• *Regional assessment of pathologically increased ventilation at baseline has the potential to prospectively identify tissue at risk for developing fibrosis.*

• *Presence and extent of pathologically increased ventilation may serve as an early imaging marker of disease activity.*

## Introduction

Idiopathic pulmonary fibrosis (IPF) is a chronic and irreversible disease with a poor prognosis with a median survival of 3 years after diagnosis and a mortality rate that surpasses many cancer types [1]. Antifibrotic medication [2, 3] was found to significantly slow down disease progression in a subset of patients and was recently also approved for non-idiopathic types of progressive fibrosis. Indication for treatment currently consists of a combination of functional decrease (based on pulmonary function testing, PFT), increased clinical symptoms, and progression of fibrosis on CT within a specific time period [4].

Recently, it was shown that abnormally high air volume changes during ventilation in lung tissue of normal density correlate with a future decrease of segmented lung volume and increase of mean lung density, both considered quantitatively accessible morphological indicators of structural changes of fibrosis. Accordingly, a correlation was seen between abnormally high ventilation and a future decrease of forced vital capacity (FVC) [5]. However, these correlations were calculated for the entire lung without assessing spatial relationships at the regional level.

In this study, we aimed to investigate whether there is a spatial correspondence between increased regional ventilation at baseline CT and morphological changes in follow-up CT scans of IPF patients. If so, this finding would help identify lung parenchyma at risk for developing fibrosis, allowing to estimate potential disease progression prospectively and more accurately than using global CT ventilation measures.

## Methods

### Study group

The Internal Review Board approved the study (No. 3649-2017). Inclusion criteria were a written patient consent, diagnosis of IPF confirmed by a multidisciplinary board, two consecutive CT scans with an interval of at least 6 months, no acute pulmonary comorbidities at the time of the CT scans, and body plethysmography measurements according to the guidelines of the European Respiratory Society within two months of the corresponding CT scans. Based on these criteria, between September 2016 and November 2020, 45 patients were consecutively included in this retrospective study.

### CT data acquisition

CT examinations were performed with a dual-source CT (Somatom Force®, Siemens Healthineers) at 90 kV/150 kV in full inspiration and maximum expiration (mean CTDI 5.23 ± 1.82 mGy for inspiration/mean CTDI 3.91 ± 1.41 mGy for expiration) in a supine position and without respiratory gating. Before CT acquisition, a training session was performed to ensure patient cooperation to the best of their abilities. The scan acquisition included intravenous contrast administration for purposes not relevant for this study. Virtual non-contrast (VNC) images were generated for both breathing positions using dedicated software (syngo.via® v 5.1, Siemens Healthineers). Previous publications had proven that VNC images are equally suited for quantitative assessment of absorption values as images acquired without intravenous contrast application [6–8]. An iterative algorithm (ADMIRE), a soft tissue kernel (Qr40), and a reconstruction interval of 1 mm were applied for CT reconstruction.

### CT data processing

The following postprocessing steps were performed:

- Segmentation

Using in-house software based on a deep learning algorithm, automatic lung segmentation was achieved [9]. Lung segmentation was performed of both, inspiration and expiration scans. Attenuation areas < − 950 HU corresponding to emphysema and areas above a threshold of − 250 HU were removed, the latter corresponding to intrapulmonary vessels and severely pathological lung parenchyma, including advanced interstitial fibrosis [10–13].

- Registration

**Fig. 1:**
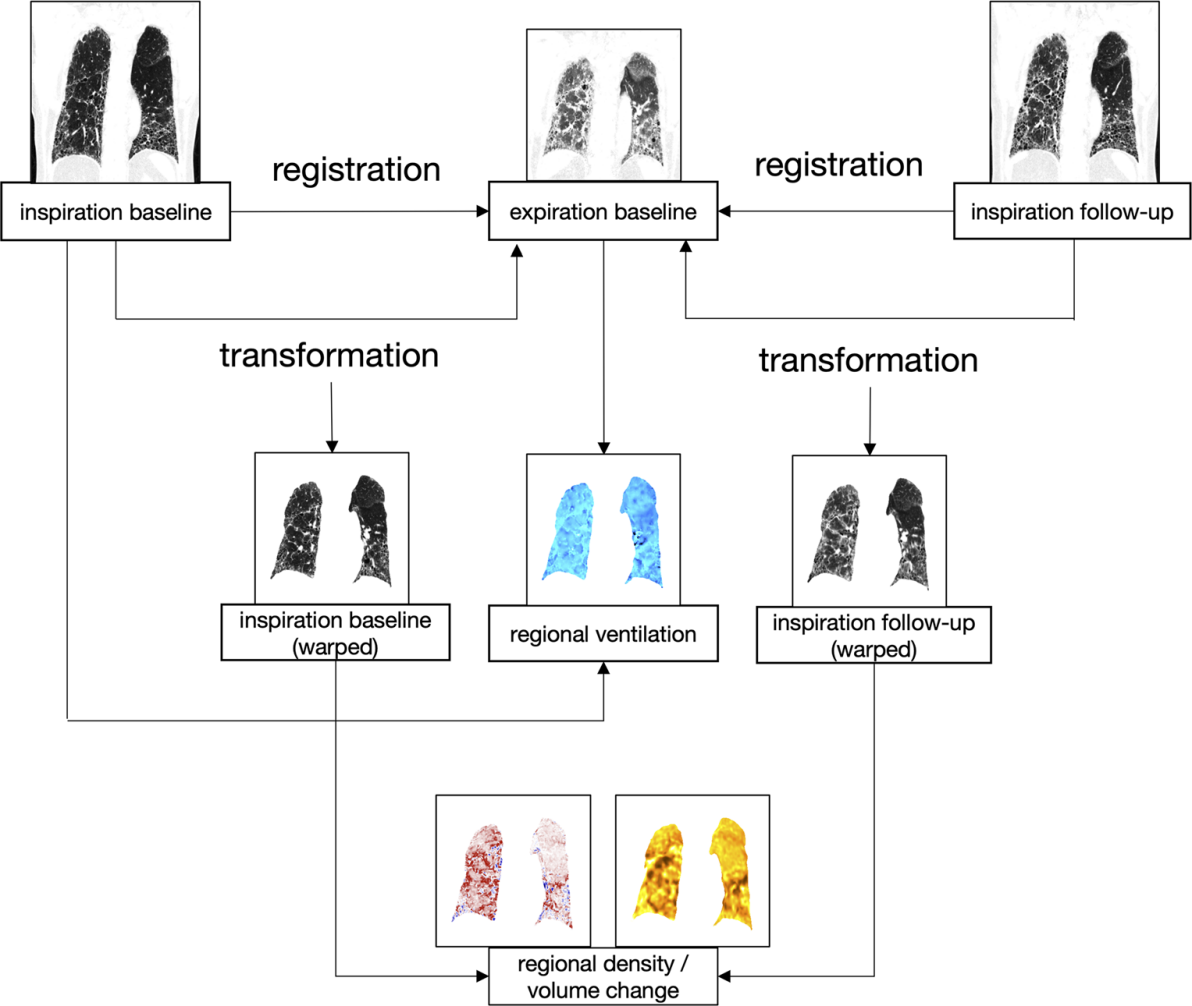
Postprocessing workflow. The baseline inspiration and the follow-up inspiration images were registered and transformed to match the baseline expiration (inspiration baseline warped and inspiration follow-up warped). The inverse Jacobian determinant was used to calculate the baseline regional ventilation from the baseline’s inspiration and expiration. A density difference image depicting temporal regional density change over time was calculated by subtracting the baseline image from the spatially aligned follow-up image. The temporal regional volume change image was generated using the Jacobian determinant of the temporal registration

The order of image processing steps is outlined in Fig. 1:

To be able to regionally (voxel-based) correlate ventilation with density and volume changes, a common “anatomical mask” had to be defined. The baseline expiration scan served as “anatomical mask.” Baseline inspiration scan was registered to the expiration scan to create the warped inspiration baseline, similarly the follow-up inspiration scan was registered to the same expiration scan to create the warped follow-up inspiration scan. The registration was done in that direction because warping a larger inspiration volume with more voxels to a smaller expiration volume with less voxels is less prone to registration errors allowing for superior voxelwise matching.

Non-linear registrations were performed using an open-source software package (ANTS, 2011, Release 2.1, Penn Image Computing and Science Laboratory).

To calculate the regional ventilation, the baseline inspiration scan was registered to the baseline expiration scan.

The baseline ventilation was calculated using the warped inspiration baseline and the expiration baseline image (Fig. 1) creating a ventilation image (in blue). To calculate the structural changes (density and volume), the warped inspiration baseline was compared to the warped inspiration follow-up creating images showing the volume (in yellow) and density change (in red/blue).

Density ranges of lung parenchyma with attenuation areas ranging from − 950 to − 600 HU on the baseline inspiration scan were defined as normal lung parenchyma (NAA). Abnormally high attenuation areas ranging from − 599 to − 250 HU (HAA) were defined as pathological lung parenchyma at baseline. Calculations of density and volume changes per ventilation intensity for NAA and HAA were done on the registered images as outlined above.

### Calculation of regional ventilation at baseline

Since CT cannot measure gas exchange directly, fractional air volume change—measured as the regional change of lung density and volume in inspiration versus expiration—has been used as a surrogate of lung ventilation [14, 15].

For registration-based calculation of regional ventilation in CT, two methods have been proposed in the literature:

The density-based method assesses the attenuation change between the end-inspiration and the end-expiration scans voxel-by-voxel. Depending on the amount of exhaled air, the density of the corresponding voxels changes proportionally between inspiration and expiration. This technique has been previously applied, e.g., to detect air trapping in small airway disease or bronchiolitis obliterans syndrome [16, 17].

The second method calculates the spatial displacement field when registering inspiration and expiration scans to indicate regional (air) volume change. The regional volume change is quantified using the Jacobian determinant.

In this study, we combined both techniques as proposed by Ding et al, because the combination of the two techniques has proven to yield superior results [18]:

We used the inverse Jacobian determinant to depict the voxelwise volume change from the inspiration scan to the expiration scan. The voxelwise attenuation difference in Hounsfield units between inspiration and expiration scans was used as correction factors.

Regional ventilation values were normalized to a range from zero to 1, corresponding to no volume change at all (0) to complete collapse in expiration (1).

$$ \mathrm{Regional}\ \mathrm{Ventilation}=1-\left(\mathrm{Inverse}\ \mathrm{Jacobian}\ast \right.\left(\frac{\mathrm{lung}\ \mathrm{density}\left(\mathrm{expiration}\right)}{\mathrm{lung}\ \mathrm{density}\left(\mathrm{inspirationWarped}\right)}\right) $$

For healthy subjects, mean ventilation values between 0.4 and 0.5 have been described for Jacobian determinant–based ventilation measurements [19, 20]. The correction factor applied by Ding et al [18] slightly increases the calculated regional ventilation to a normal value around 0.5.

### Serial structural change

Structural change over time was defined as a regional change of density and volume on CT. Following temporal registration, a voxelwise calculation of the regional density difference (in Hounsfield units, HU) was performed on the paired baseline and the follow-up scan (pos. values = density increase, neg. values = density decrease). The temporal regional volume change was calculated using the deformation field (Jacobian determinant image) of the non-linear registration between the baseline and the follow-up scan, calculating the lung volume change per voxel.

Images were calculated per CT voxel with transversal dimensions of 512 × 512 voxels. Applying a field of view of 35 cm, the voxel size was 0.68 × 0.68 × 1 mm.

### Statistical analysis

To assess the spatial correlation of increased ventilation at baseline and structural change at follow-up, normalized regional ventilation, ranging from zero to 1, was divided into four categories: 0–< 0.25 (C1), 0.25–< 0.5 (C2), 0.5–< 0.75 (C3), and 0.75–1 (C4). Previous data suggest that the range of normal ventilation is centered around 0.5. Therefore, C2 was considered low-to-normal ventilation, C3 moderately increased ventilation, and C4 severely increased ventilation.

The statistical analysis was performed separately for NAA (ranging from − 950 to − 600 HU) and HAA (ranging from − 599 HU to − 250 HU).

Lower attenuation areas (LAA, < − 950 HU), representing severe emphysema or air in cysts, and higher attenuation areas (> − 250 HU) representing highly pathologic areas were excluded. These thresholds were determined on the basis of published data [10–13].

We calculated mean density change and mean volume change in the serial CT scans separately for the four ventilation categories (C1–C4) at baseline. The significance of the differences in density and volume changes between the four ventilation categories was assessed using paired *t*-tests with a significance level of *p* ≤ 0.05. The analysis was performed separately for NAA and HAA.

Statistical analysis was carried out using SPSS Statistics 25 (IBM) and MATLAB R2019a (MathWorks). The open-source package Fiji (https://imagej.net/Fiji) was used for visualization purposes.

## Results

### Study group

Four patients were excluded from ventilation analysis due to inaccurate registration (misalignment > 2 mm). Thus, the final study group consisted of 41 patients.

The mean time between baseline and follow-up CT was 15 ± 7 months. PFT data were obtained within 14 ± 14 days of the baseline CT and within 15 ± 17 days of the follow-up CT.

Patient population characteristics are depicted in Table 1.

**Table 1:** Patient characteristics. Most patients were elderly males with a mean age of 73 years and a mean decline of forced vital capacity (FVC%) of 8% between baseline and follow-up. Continuous variables are stated as mean and standard deviation (SD), and categorical variables are stated as *n* unless indicated otherwise

No. of patients	41
Sex—*n*	
- Male	36
- Female	5
Age (years)	73 ± 10
Smoking status	
- Never	16
- Former	24
- Current	1
FVC % baseline	72 ± 18
FVC % follow-up	64 ± 16

### Regional ventilation at baseline

**Fig. 2:**
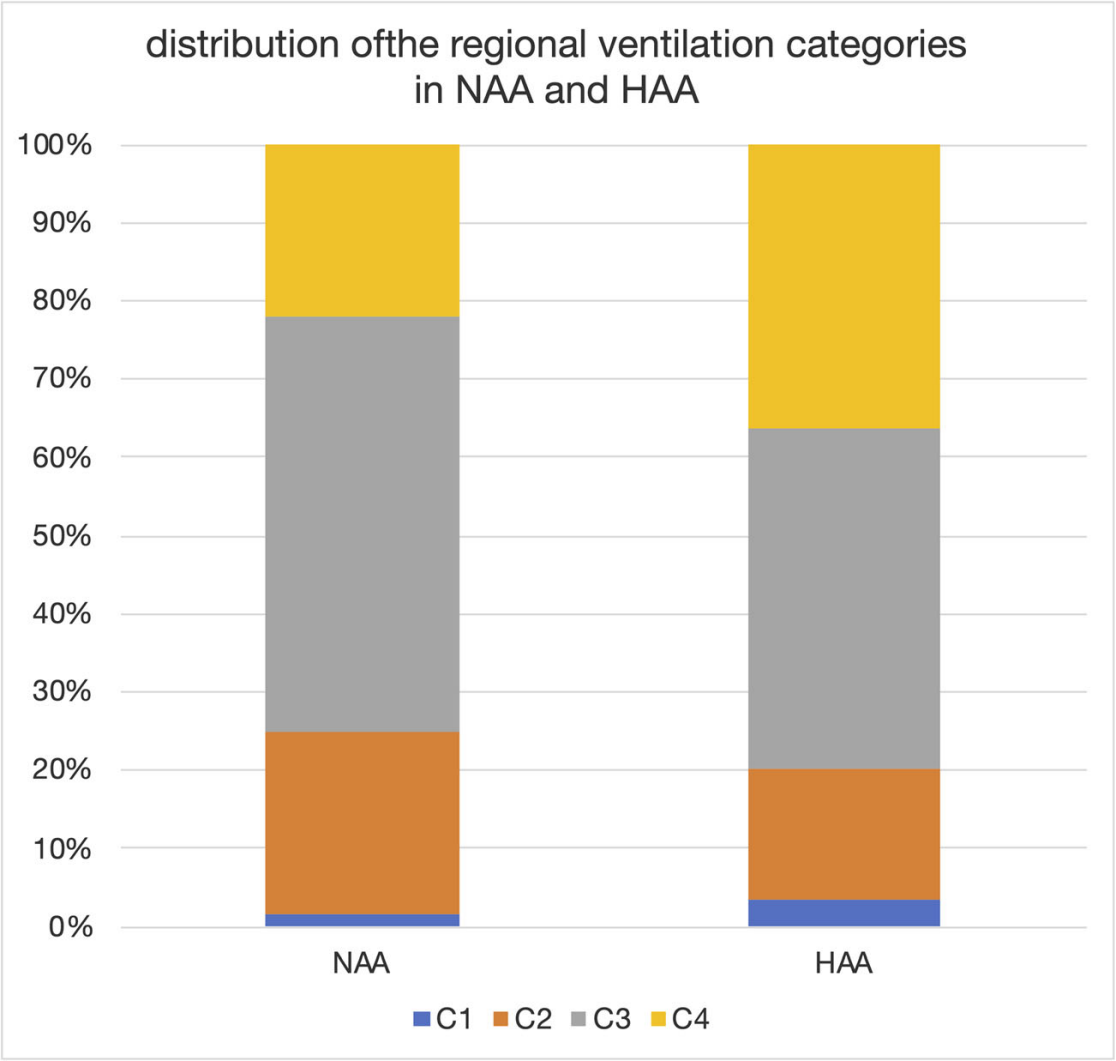
Frequency distribution ventilation C1–C4. Frequency distribution of the regional ventilation categories in the different tissue areas, normal attenuation areas (NAA), and high attenuation areas (HAA) at baseline as a percentage of lung volume

The volume fractions of the four ventilation categories are shown in Fig. 2.

Regional ventilation < 0.25 (C1) only amounted to 1.6% and 3.3 % of the lung volume in NAA and HAA, respectively, and was mainly located at the bronchial and vascular interface anatomically corresponding to the peribronchovascular interstitium. Therefore, these volume fractions were considered not taking part in ventilation and were ignored for the subsequent data analysis.

HAA contained a larger volume fraction of severely increased ventilation (C4) at baseline compared to NAA (22.1% versus 35.7%). Correspondingly, the volume fraction of low-to-normal or moderately increased ventilation (C2 and C3) was larger in NAA than in HAA (23.2% vs. 16.6% for C2, and 53.0% vs. 43.2% for C3, respectively).

### Serial structural changes

Density and volume changes over time averaged over all patients and as a function of baseline ventilation are depicted in Table 2.

**Table 2:** Mean temporal density and volume change ± SD depending on the ventilation at baseline in normal attenuation areas (NAA) and high attenuation areas (HAA)

Ventilation	Pooled mean density change ± SD (HU)		Pooled mean volume change ± SD (%)	
Baseline	Follow-up		Follow-up	
	NAA	HAA	NAA	HAA
0.25–0.50	23.78 ± 33.07	− 34.35 ± 38.31	+ 2.25 ± 4.99	+ 1.37 ± 4.72
0.50–0.75	31.69 ± 33.62	− 11.50 ± 37.39	− 0.80 ± 3.53	− 0.79 ± 5.80
0.75–1.00	57.68 ± 41.24	15.14 ± 48.96	− 3.74 ± 5.40	− 0.78 ± 7.17

The change in density—as a function of voxel-based baseline ventilation—was different for NAA and HAA.

In NAA, density showed a positive correlation with ventilation over the entire ventilation range. The higher the ventilation at baseline, the higher the increase in density at follow-up. The increase in density was about twice as high in the area of severely increased ventilation (C4) as compared to normal or moderately increased ventilation (C2 or C3).

In HAA, an increase of density was observed only in regions with severely increased ventilation (C4), while a decrease in mean density was seen in the remaining two areas of lower ventilation (C2 and C3)

In both NAA and HAA, the difference of mean density change reached statistical significance (*p* < 0.001) in the area of severely increased ventilation (C4) compared to the remaining areas. In general, the density changes in HAA were more heterogeneous than in NAA indicated by the larger SD (Table 3).

**Table 3:** Results of statistical analysis for density change. Paired *t*-tests comparing the mean temporal density change in the top category of ventilation (0.75 and above) against lower ventilation values at baseline in normal attenuation areas (NAA) and high attenuation areas (HAA)

	Paired differences between ventilation categories (HU)				*p* value
	Mean difference	Std. deviation	95% CI of the difference		
			Lower	Upper	
Mean density change NAA (HU)					
C4 vs C2	33.90	23.04	26.63	41.18	0.000
C4 vs C3	25.99	16.59	20.76	31.23	0.000
Mean density change HAA (HU)					
C4 vs C2	45.49	45.04	31.28	59.71	0.000
C4 vs C3	22.65	26.73	14.21	31.08	0.000

For the second parameter, serial volume change, in general, volume loss was seen in areas of increased baseline ventilation (C3 and C4), and a volume increase was seen in low-to-normal ventilation (C2), in both NAA and HAA (Table 2).

In NAA, the difference of volume change reached significance between severely increased ventilation C4 and both areas of lower ventilation (C2 and C3, both *p* < 0.001)

In HAA, the difference of volume change reached significance only between severely increased ventilation C4 and low-to-normal ventilation (C2, *p* = 0.025) (Table 4).

**Table 4:** Results of statistical analysis for volume change. Paired *t*-tests comparing the mean temporal volume change in the top category of ventilation (0.75 and above) against lower ventilation values at baseline in normal attenuation areas (NAA) and high attenuation areas (HAA)

	Paired differences between ventilation categories (HU)				*p* value
	Mean difference	Std. deviation	95% confidence interval of the difference		
			Lower	Upper	
Mean volume change NAA (%)					
C4 vs C2	− 5.99	7.91	− 8.48	− 3.49	0.000
C4 vs C3	− 2.94	4.36	− 4.31	− 1.56	0.000
Mean volume change HAA (%)					
C4 vs C2	− 2.15	5.90	− 4.01	− 0.29	0.025
C4 vs C3	0.01	2.66	− 0.83	0.84	0.988

**Fig. 3:**
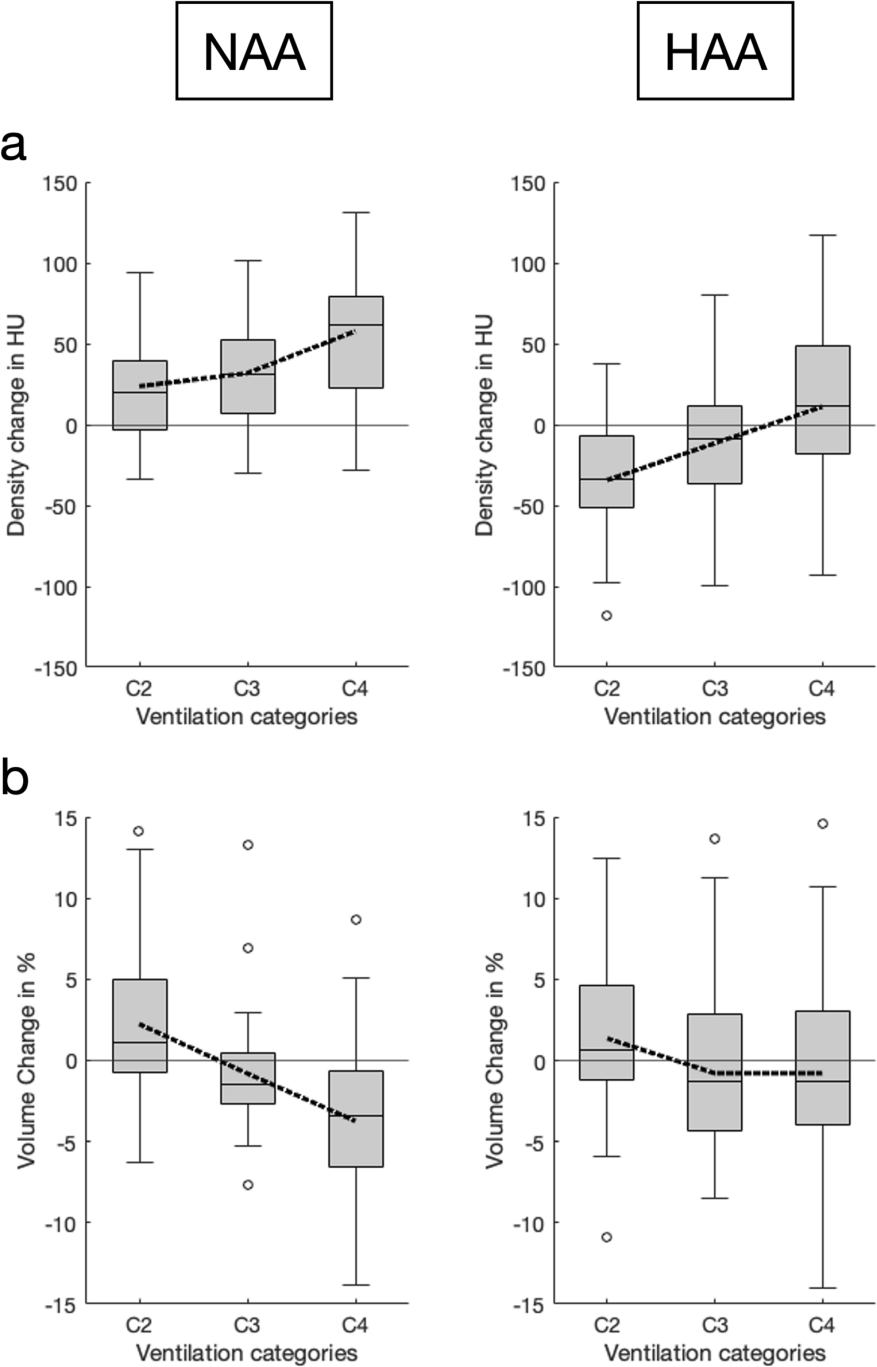
Density and volume changes depending on baseline ventilation. Box charts showing the temporal density (**a**) and volume changes (**b**) in normal attenuation areas (NAA) and high attenuation areas (HAA) averaged over all patients as a function of baseline regional ventilation (C2–C4). Additionally, the mean values of the density and volume change are superimposed as dotted line plots

Figure 3 illustrates the spatial correspondence of baseline ventilation and subsequent density and volume changes, namely density increase and volume loss with increasing baseline ventilation. As an exception, in HAA, volume loss was relatively small from C3 to C4.

### Case examples

**Fig. 4:**
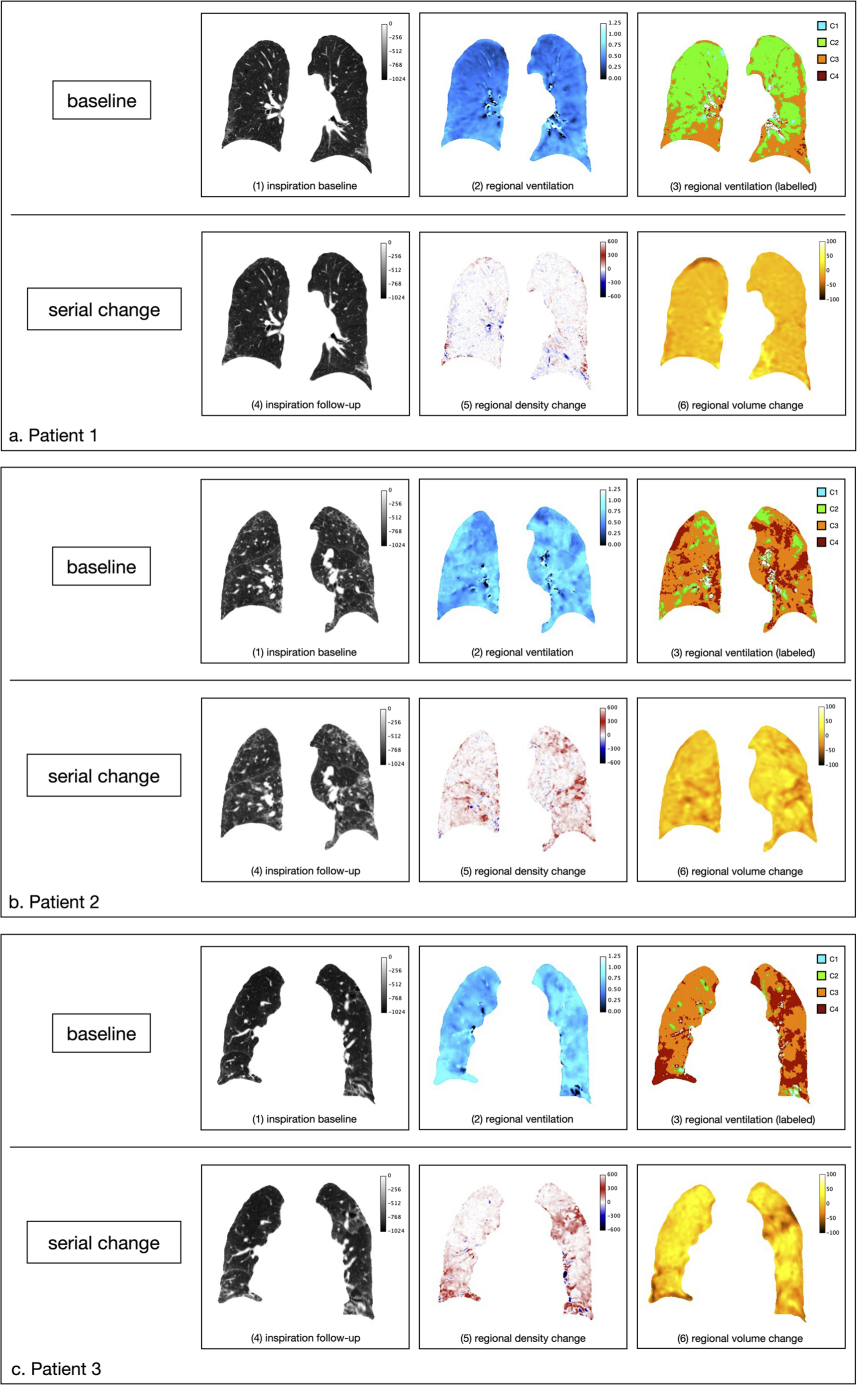
Three case examples. The upper row shows the baseline inspiration (1), regional ventilation at baseline (2), and regional ventilation at baseline labeled into four categories encoded by different colors (3). Green indicates areas of low-to-normal baseline ventilation (C2), while orange (C3) and dark red (C4) indicate areas of normal to moderately and severely increased baseline ventilation, respectively. The bottom row per plot shows the follow-up inspiration scan warped to the baseline CT (4), the corresponding regional density (5), and the regional volume change images (6) again warped to the baseline expiration scan (see also Fig. 1). The color-coded scales in (1) and (4) represent HU values, in (2) normalized regional ventilation values, in (5) HU changes and in (6) volume changes in % values, in (2) normalized regional ventilation values, in (5) HU changes and in (6) volume changes in %

Exemplary CT and ventilation images of three patients are presented in Fig. 4.

Patient 1 (interval 12.3 months, no FVC% decline) shows mainly C2 ventilation (mean regional ventilation: 0.46, C2: 60%, C3: 33%, C4: 3%) and only minor changes in density and volume over time.

Patient 2 (interval 16.4 months; FVC% decline by 10%) shows heterogeneous ventilation with areas of increased and normal regional ventilation (mean regional ventilation: 0.64, C2: 12%, C3: 68%, C4: 20%).

Patient 3 (interval 16.0 months; FVC% decline by 16%) shows more prominently increased ventilation with mainly C3 and C4 ventilation (mean regional ventilation: 0.73, C2: 4%, C3: 49%, C4: 47%).

Areas of increased ventilation at baseline (C4: dark red labels) spatially correspond to areas with major density (dark red) and volume change (dark orange).

Though in some patients a generally increased baseline ventilation was observed, the highest ventilation values were mostly found in the basal and peripheral lung, thus in areas of typically seen fibrotic changes. In this feasibility study, we only qualitatively established a spatial correlation between baseline ventilation and progressive fibrosis; no patient-based quantitative analysis was performed.

**Fig. 5:**
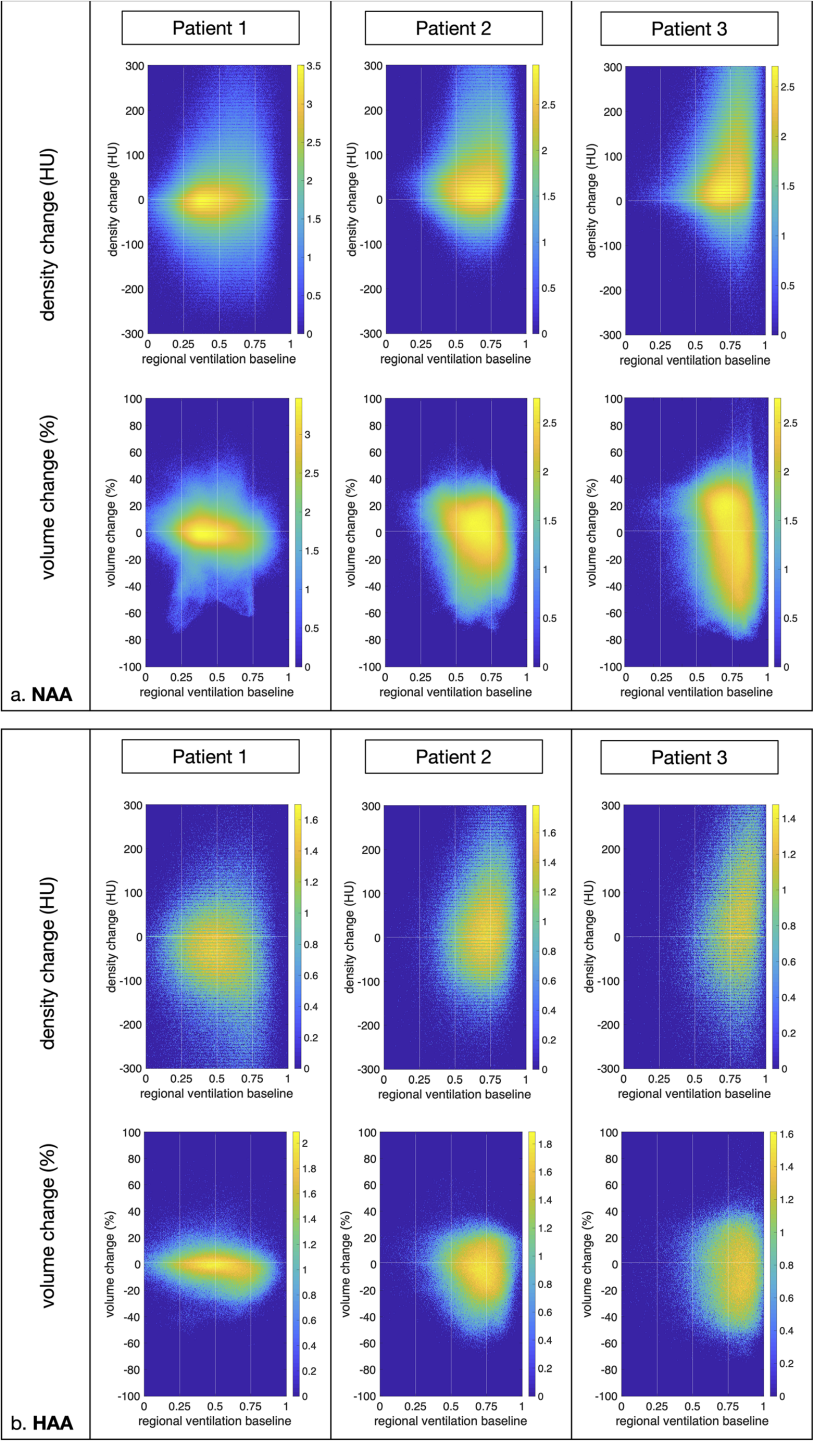
Scatterplots depicting density and volume change as a function of ventilation. Scatterplots showing voxelwise ventilation-density change and ventilation-volume change of three patients with varying baseline ventilation. Increased regional ventilation (C3 and C4) leads to a higher density increase and volume loss than C2. *x*-axis: baseline regional ventilation; *y*-axis upper row: temporal density change (HU)/*y*-axis bottom row: temporal volume change. **a** Ventilation-density/volume change in normal attenuation areas (NAA). **b** Ventilation-density/volume change in high attenuation areas (HAA). The frequency distribution is normalized and color-coded, applying a log scale from blue to yellow

Voxelwise scatterplots of the same three patients show the spatial correlation between ventilation at baseline and temporal density/volume change on follow-up (Fig. 5).

In patient 1, the ventilation values are mainly found in C2 and only minor temporal changes of density or volume in the follow-up as indicated by the symmetric cloud of values.

In the other two patients, the ventilation values were higher, mostly lying in C3 in patient 2 and to a substantial part in C4 in patient 3, resulting in a right shift of the cloud. The right shift goes parallel with an increasing asymmetry of the data cloud towards a density increase and volume loss.

## Discussion

A previous study had revealed a correlation between increased ventilation and future density increase and volume loss for the whole lung. The aim of this study was to investigate whether there is a spatial association between voxel-based increased baseline ventilation and structural changes on follow-up CT that—if found—would be suggestive for a causative correlation.

In this study, a number of assumptions were made that were supported by previous publications:

We used a density-corrected volume change in inspiration versus expiration per voxel as a surrogate for ventilation: the higher the normalized regional ventilation (on a scale between 0 and 1.0), the higher the volume difference during the respiratory cycle up to the extreme of complete alveolar collapse [21].

Secondly, structural changes as volume loss and density increase over time (in the absence of pulmonary comorbidities) were considered signs of progressive fibrosis [22, 23]. To get an insight, whether these changes occurred predominantly in normally aerated lung parenchyma or in the pathological lung parenchyma, areas of normal attenuation (NAA) and of increased attenuation (HAA) at baseline were analyzed separately.

Results show that there is not only a global but also a spatial correlation between increased ventilation on CT at baseline and structural changes corresponding to fibrosis progression on follow-up CT.

The higher the baseline ventilation in the normal lung parenchyma, the higher the density increase and volume loss.

Traction forces related to respiration are highest in the peripheral and basal lung, also under physiological conditions. The injury occurs only when surfactant dysfunction—inherited or acquired—fails to protect the alveolar parenchyma from tractional injury resulting in alveolar collapse [21, 24]. Subsequent collapse induration has been proposed as the primary pathway for IPF [25]. Based on that, we interpret the areas with moderately and especially severely increased ventilation (C4) as areas of potential tractional injury [26]. Increased ventilation did not necessarily lead to subsequent structural changes. However, vice versa, significant changes of volume and density almost exclusively occurred in areas of increased ventilation (C3 and C4). Based on our findings, we, therefore, propose that areas of pathologically increased ventilation on CT have the potential to serve as an imaging marker for “tissue at risk” to develop fibrosis subsequently; however, larger and prospective studies are needed to support this concept.

The relation between baseline ventilation and subsequent structural changes was different in normal attenuation areas (NAA) compared to areas with pathological attenuation (HAA). In NAA, the relationship between ventilation and structural changes was consistent. Increased baseline ventilation was accompanied by an increase in regional density and volume loss. The degree of volume loss was more pronounced in NAA than in HAA. In HAA, the relationship between ventilation and structural changes was more heterogeneous. HAA represents regions with higher density than normal lung parenchyma, thus in the absence of infection areas with interstitial changes of variable degree and type. It has to be noted that HAA may include reversible as well as irreversible changes.

In HAA, only in areas with highly pathological ventilation (C4) density and volume changes reached significance compared to the remaining areas. Volume loss and density change in areas of C4 were interpreted as progressive fibrosis similarly as we observed in NAA. However, structural changes in already existing fibrosis are more complex. Fibrosis-induced traction causes regionally heterogeneous density and volume changes with density increase and volume loss in fibrosis on one side accompanied by volume expansion and density decrease of adjacent regions by traction and bronchiectasis on the other side [26, 27]. In that respect, we observed volume expansion in areas bordering regions with high ventilation/progressing fibrosis suggesting a traction mechanism. In our relatively small patient group, structural changes in areas of normal and moderately increased ventilation reached no significance in HAA, and also included volume increase and density decrease. Whether lack of significance is due to the small numbers or due to the absence of correlation, remains open.

The study has several limitations.

The study setup was retrospective, and the study group was small and heterogeneous with respect to the interval time between serial CTs. The small study group also explains the relatively large standard deviations. Our study, therefore, may be considered a feasibility study. More patients with various degrees of disease progression need to be evaluated to prove the concept. However, the fact that we still observed highly significant results in this rather limited patient group seems to underline the causative relationship between severely increased ventilation and future structural changes.

Dual registration was required to align baseline inspiratory, and expiratory baseline and CT scans at two points in time with a single anatomic reference scan.

This is technically very demanding and is a potential source for inaccuracies in calculating the Jacobian determinant for the assessment of voxel-based ventilation and volume change in the serial scans.

Ventilation is affected by patient position, gravity effects [28], and breathing depth, with the respective impact unknown. Further evaluations are required to assess the effects of position and respiratory depth on the reproducibility of the measurements. CT scans had been acquired after contrast media injection that was done for different study purposes. In this study, therefore, virtual non-contrast (VNC) images were used, which might have influenced the quantification measures to a certain extent. Since all patients had been obtained with the same technique, we consider this factor as small and not influential on our results [6, 29].

## Conclusion

The voxel-based analysis suggests a spatial correspondence between regional ventilation and morphological changes over time. Regions with pathologically high ventilation may serve as an indicator of tissue at risk in the baseline CT for developing structural changes over time indicative of progression of fibrosis.

## Abbreviations

FVC: Forced vital capacity
HAA: High attenuation areas
HU: Hounsfield units
IPF: Idiopathic pulmonary fibrosis
LAA: Low attenuation areas
NAA: Normal attenuation areas
PBV: Pulmonary blood volume
PFT: Pulmonary function test
VNC: Virtual non-contrast

## Declarations

### Guarantor

The scientific guarantor of this publication is Hoen-oh Shin.

### Statistics and biometry

No complex statistical methods were necessary for this paper.

### Study subjects or cohorts overlap

Some study subjects or cohorts have been previously reported in Scharm, S.C., Vogel-Claussen, J., Schaefer-Prokop, C. et al. Quantification of dual-energy CT-derived functional parameters as potential imaging markers for progression of idiopathic pulmonary fibrosis. *Eur Radiol* 31, 6640–6651 (2021). https://doi.org/10.1007/s00330-021-07798-w

### Methodology

• retrospective

• observational

• performed at one institution
